# Composite Hydrogels with Engineered Microdomains for Optical Glucose Sensing at Low Oxygen Conditions

**DOI:** 10.3390/bios7010008

**Published:** 2017-01-22

**Authors:** Lindsey R. Bornhoeft, Aniket Biswas, Michael J. McShane

**Affiliations:** 1 Department of Biomedical Engineering; Texas A&M University, College Station, TX 77843, USA; lrbornhoeft@tamu.edu (L.R.B.); aniketbiswas@tamu.edu (A.B.); 2 Department of Materials Science and Engineering, Texas A&M University, College Station, TX 77843, USA

**Keywords:** bioresponsive hydrogel, enzymatic, phosphorescence, alginate, microparticle, layer-by-layer, porphyrin

## Abstract

There is a growing need for advanced tools that enable frequent monitoring of biomarkers for precision medicine. In this work, we present a composite hydrogel-based system providing real-time optical bioanalyte monitoring. The responsive material, alginate-in-alginate (AnA), is comprised of an alginate hydrogel with embedded bioactive, nanofilm-coated phosphorescent microdomains; palladium tetracarboxyphenylporphyrin serves as an optical indicator, glucose oxidase as a model enzyme, and layer-by-layer deposited polyelectrolyte multilayers (PEMs) as the diffusion barrier. Glutaraldehyde crosslinking of the nanofilms resulted in a dramatic reduction in glucose diffusion (179%) while oxygen transport was not significantly affected. The responses of the AnA hydrogels to step changes of glucose at both ambient and physiological oxygen levels were evaluated, revealing controlled tuning of sensitivity and dynamic range. Stability, assessed by alternately exposing the responsive AnA hydrogels to extremely high and zero glucose concentrations, resulted in no significant difference in the response over 20 cycles. These AnA hydrogels represent an attractive approach to biosensing based on biocompatible materials that may be used as minimally-invasive, implantable devices capable of optical interrogation. The model glucose-responsive composite material studied in this work will serve as a template that can be translated for sensing additional analytes (e.g., lactate, urea, pyruvate, cholesterol) and can be used for monitoring other chronic conditions.

## 1. Introduction

Chronic diseases (CDs) such as cancer, kidney failure, and diabetes affect millions worldwide. Advances in management and healthcare of individuals suffering from CDs have led to a significant reduction in CD related patient mortality and, consequently, an increased prevalence of CD in the global population [1,2]. Additionally, effective pharmaceuticals such as antibiotics and vaccinations created to treat and prevent infectious diseases have produced a population with a much longer life expectancy than those of previous generations [3]. These concurrent circumstances are synergistically leading to an aging population that will place a heavy burden on healthcare systems globally; this will necessitate paradigm shifts in self-management by patients, including the development of methods and technology for self-monitoring of important biomarkers [4,5]. With user-friendly tools that quantify critical markers of CD such as lactate [6], urea [7], and glucose [8], patients will be enabled to better monitor and control the progression of their disease state and therefore be empowered to improve their overall well-being and quality of life.

However, a simple, minimally-invasive, continuous bioanalyte monitoring system still remains beyond the capabilities of devices on the market today. Devices requiring discrete blood or serum sampling are incapable of detecting all major fluctuations that occur throughout the day, often missing relevant highs and lows. Compounding the limitations of the technology are the social and practical aspects such as low patient compliance and the risk of infection resulting from painful, skin-perforating sampling methods. Fully non-invasive wearable devices have attempted to overcome these limitations by measuring analytes (e.g., pH [9,10], electrolytes [11,12], lactate [13,14], or glucose [15,16]) in excreted and secreted fluids. Unfortunately, these non-invasive devices tend to show limited correlation to in vivo analyte concentrations and are highly variable due to environmental and lifestyle factors such as eating, drinking, sweating, and exercising. Minimally-invasive, electrochemical sensors (e.g*.*, continuous glucose monitoring systems) have provided both analyte specificity and continuous monitoring; these devices have recently met accuracy benchmarks sufficient to significantly reduce the number of daily finger prick samples [17], but they remain cumbersome for users due to limited operating lifespans and the need for replacement/re-insertion of new sensors every 7–10 days.

Fully-implantable bioresponsive materials offer the potential to overcome these issues; however, devices proposed for implantation carry stringent requirements. Any sensing material that is completely implanted should satisfy the following basic criteria: (1) soft biomaterial possessing a small form factor and mechanical properties similar to the surrounding tissue [18]; (2) high-fidelity response over the biologically relevant range [19]; and (3) long-term stability [20]. Insensitivity to electromagnetic interference (EMI) and low/zero-power operation with facile recharging are additional desirable properties [21]. To meet these requirements in an approach that offers the additional advantage of avoiding the use of implanted electronics, our group has recently developed bioresponsive materials that respond to lactate and glucose by covalently immobilizing oxidoreductase enzymes and an oxygen-sensitive metalloporphyrin dye within biocompatible hydrogel matrices [22,23]. These unique materials transduce chemical concentration into optical information (phosphorescence intensity or lifetime). While these materials were found to exhibit the desired response characteristics in benchtop testing, it was observed that the response often saturated at relatively low analyte concentrations when used in vivo; this undesired outcome has been attributed primarily to low tissue oxygen levels which have been found to be ≈3 times lower than air-equilibrated aqueous solutions [24,25]. These low oxygen concentrations disrupt the function of the bioresponsive material because they were designed and lab-proven based on a diffusion-reaction equilibrium expecting higher oxygen concentrations. Modification of the materials to address this issue requires drastic reduction of analyte flux; this is essential because enzymatic bioresponsive materials require a delicate balance between analyte diffusion and reaction to produce a signal that varies sensitively over the entire analyte concentration range of interest [26,27]. The use of polyelectrolyte multilayers (PEM) to form responsive microdomains dispersed in hydrogel matrices to control glucose diffusion was recently demonstrated as an effective means to “tune” the optical response; these responsive materials are capable of preferentially reducing glucose flux while not affecting oxygen diffusion kinetics [28,29]. However, the relatively low enzyme stability in the hollow microdomains of these materials was insufficient to support use in long-term sensing applications. In contrast, ionically-crosslinked alginate, a polysaccharide with mild encapsulation and gelation techniques, was chosen as an improvement in the material to enhance enzyme stability [30,31].

In this work, the same principle of transport control via nanofilm coatings on microdomains mentioned above was adapted for application to alginate microparticles that were subsequently embedded in alginate hydrogels. We hypothesized that the sensitivity to different oxygen concentrations could be influenced by the composition of the nanofilm coating and that there would be a substantial increase in stability by protecting and immobilizing enzymes within alginate microparticles. This design was expected to leverage the advantages of different systems studied in our previous work, providing enzyme stability along with the capability to fine-tune the optical response of the material for different applications/environments; together, these properties give a level of engineering control over the sensing figures of merit that, to our knowledge, has not been demonstrated before.

## 2. Materials and Methods

### 2.1. Reagents and Materials

All chemicals were reagent-grade and used without further purification. Alginic acid sodium salt (250 cP, 2% @ 25 °C), buffer salts (TRIS, 2-(*N*-morpholino) ethanesulfonic acid), calcium carbonate (CaCO_3_), 2,2,4-trimethylpentane (isooctane), poly(allylamine hydrochloride) (PAH, average MW 15 kDa), poly(sodium-4-styrenesulfonate) (PSS, average MW 70 kDa), dimethyl sulfoxide (DMSO), and glutaraldehyde (grade II, 25% in deionized water) were obtained from Sigma Aldrich (St. Louis, MO, USA). β-d-glucose and calcium chloride (CaCl_2_) were obtained from Macron Fine Chemicals (Center Valley, PA, USA). The surfactants sorbitan trioleate (SPAN 85) and polyoxyethylene sorbitan trioleate (TWEEN 85) were obtained from Tokyo Chemical Industry Company (Tokyo, Japan) and Sigma Aldrich, respectively. Pd-meso-tetra(4-carboxyphenyl) porphyrin (PdTCPP) was obtained from Frontier Scientific (Logan, UT, USA). Glucose oxidase (284 U/mg, *Aspergillus niger*) and catalase (6500 U/mg, bovine liver) were obtained from BBI Solutions (Cardiff, UK) and Calzyme Laboratories, Inc. (San Luis Obispo, CA, USA), respectively.

### 2.2. Alginate Microparticle Synthesis

Alginate microparticles containing encapsulated PdTCPP, glucose oxidase (GOx), and catalase (Cat) were synthesized following a previously reported emulsion method with minor modifications [32]. Briefly, 5 mL of aqueous solution containing 3 w/v % alginic acid, 500 μL of 1:1 molar ratio GOx (11.7 mg) and Cat (18.3 mg) in 50 mM TRIS buffer (pH 7.2), and 500 μL of 10 mM PdTCPP in DMSO were added drop-wise and emulsified in a solution containing 10.8 mL isooctane (7.5 g) with 170 μL SPAN 85 (0.16 g) using a homogenizer (S25N18G dispersing tool, IKA T25, Wilmington, NC, USA) operating at 8000 rpm for 2 min. A second surfactant solution containing 1.5 mL isooctane (1.0 g) with 96 μL TWEEN 85 (0.10 g) was added and homogenized at the same speed for 1 min. For external gelation of alginate microparticles, 4 mL of 10 w/v % CaCl_2_ solution were added, and the emulsion was stirred gently in a round bottom flask for 20 min. Alginate microparticles were washed with deionized water and collected after centrifugation (2000 g for 2 min).

### 2.3. Layer-by-Layer (LbL) Deposition on Alginate Microparticles

Nanofilms were deposited on alginate microparticles by alternately adding 1 mL of 20 mg/mL PAH (pH 8) and 1 mL of 20 mg/mL PSS (pH 7.2) to microparticles and washing between each deposition with 10 mM TRIS buffer (pH 8 and pH 7.2, respectively) until the desired number of layers were applied. Studies of diffusion inhibition by the nanofilms have been previously performed to evaluate the relationship between the number of layers and the flux of glucose and oxygen [28]; based on those findings, 10 bilayers were used for this work. Covalent crosslinking of the PAH amine groups was performed by mixing 4.4 mg of LbL-coated alginate microparticles with 20 mL of 3 M glutaraldehyde and gently stirring for 30 min at room temperature. Particles were washed with deionized water and stored at 4 °C in a solution containing 10 mM TRIS (pH 7.2) and 10 mM CaCl_2_.

### 2.4. AnA Fabrication: Alginate Microparticles Embedded in Alginate Hydrogel

**Figure 1:**
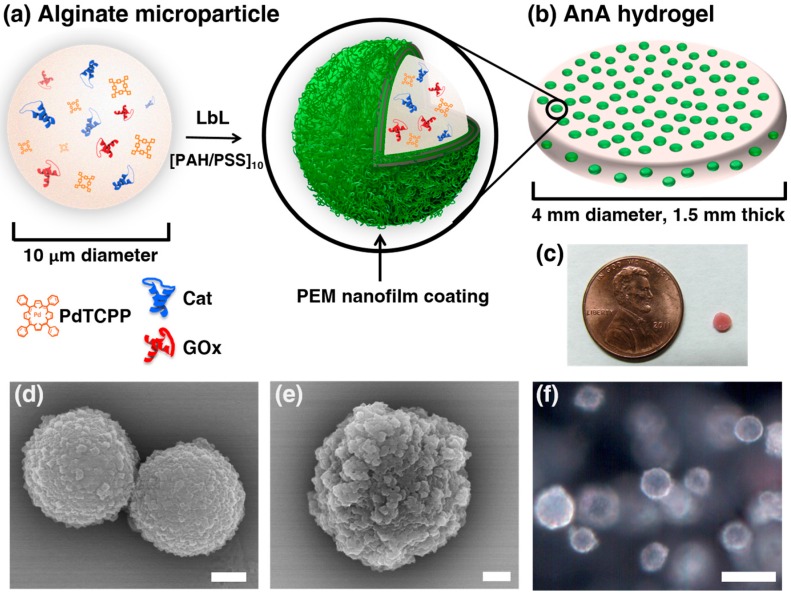
Alginate in alginate (AnA) hydrogels: composition and microscopic imaging. (**a**) Illustration of an alginate microparticle containing Pd-meso-tetra(4-carboxyphenyl) porphyrin (PdTCPP), glucose oxidase (GOx), and catalase (Cat) coated with 10 bilayers of poly(allylamine hydrochloride) (PAH) and poly(sodium-4-styrenesulfonate) (PSS); (**b**) AnA hydrogel with embedded polyelectrolyte multilayer (PEM) coated alginate particles; (**c**) Photograph of AnA hydrogel next to a penny; SEM micrograph of (**d**) uncoated and (**e**) coated alginate microparticles, respectively. Scale bars = 1 μm for both micrographs; (**f**) Darkfield optical image of the AnA hydrogel containing alginate microparticles distributed throughout an alginate matrix. Scale bar = 10 μm.

The alginate hydrogel fabrication process was based on a previously reported internal gelation method with minor modifications [33]. Briefly, alginate microparticles (75 μL, 8.8 mg) were mixed with 200 μL of 3 w/v % alginate and 25 μL of 33.3 mg/mL CaCO_3_. Dissolution of CaCO_3_ was initiated by the addition of 100 μL of 10 mM MES buffer (pH 6.1). The solution was vortexed and quickly transferred to a 1.5 mm-thick Teflon and glass mold and allowed to gel for 15 min. AnA hydrogels were then punched into discs (4 mm diameter, 1.5 mm thick, Figure 1c) using a biopsy punch. All materials were stored in a solution containing 10 mM TRIS (pH 7.2) and 10 mM CaCl_2_ and allowed to equilibrate overnight before use.

### 2.5. Diffusion Measurements

**Figure 2:**
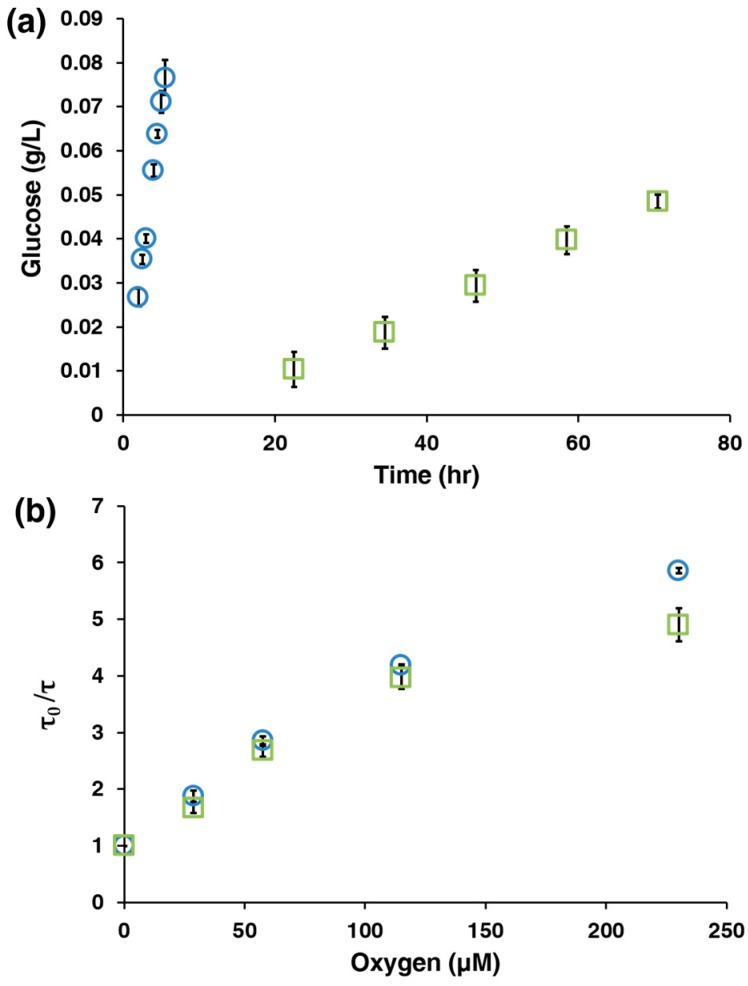
(**a**) Plot showing the difference in the rate of glucose permeation through 10 bilayers of PAH/PSS without (blue **○**) and with (green □) glutaraldehyde crosslinking; (**b**) The phosphorescence lifetimes of AnA hydrogels containing 10 bilayers of PAH/PSS plotted against the oxygen concentrations for particles without (blue ○) and with (green □) glutaraldehyde crosslinking. Error bars for both plots represent 95% confidence intervals for three separate samples.

LbL polyelectrolyte nanofilms were deposited on 0.2 μm filters (Anapore) by alternately adding 1 mL of 20 mg/mL PAH (pH 8) and 1 mL of 20 mg/mL PSS (pH 7.2) to the filters while washing between each deposition with 10 mM TRIS buffer (pH 8) until the desired number of layers were applied. Covalent crosslinking of the nanofilms was performed by adding 1 mL of 3 M glutaraldehyde for 30 min at room temperature followed by rinsing with deionized water. The nanofilm-coated Anapore filters were placed in a side by side permeate chamber (Permegear Inc., Hellertown, PA, USA) with the feed chamber containing 7 mL of a 1 g/L glucose solution and the permeate chamber containing 7 mL of a 10 mM TRIS buffer (pH 7.2). Samples were taken at regular time points from both chambers and analyzed with a Biochemistry Analyzer (Model 2700, YSI, Yellow Springs, OH, USA). The change in glucose concentration over time (d*C/*d*t*) was calculated using linear regression (Figure 2a).

### 2.6. Oxygen and Glucose Challenges

AnA hydrogels were immobilized in a custom-built flowcell previously reported elsewhere [28]. Oxygen challenges were performed by exposing the AnA hydrogels to varying dissolved oxygen concentrations (0–206.8 μM, 37 °C). The oxygen concentration was adjusted using mass flow controllers (1179A, MKS Instruments, Inc., Andouver, MA, USA). Glucose challenges were performed by exposing the AnA hydrogels to varying physiologically relevant glucose concentrations (0–400 mg/dL in 10 mM TRIS with 10 mM CaCl_2_, 37 °C) at fixed oxygen concentrations (206.8 μM for ambient and 70 μM for interstitial oxygen concentration) [24]. The oxygen concentration was regulated by a vacuum degassing chamber (9000–1118, Systec) and vacuum pump (9000-1472, Systec). All optical measurements were recorded using a custom-built time domain phosphorescence lifetime measurement instrument with a 530 nm excitation source described elsewhere [22,23]. The combination of the AnA hydrogel (bioresponsive material) with the lifetime measurement unit (detector) will be referred to as the “sensing system”. The glucose challenge data of phosphorescence lifetime versus concentration were fit using a sigmoidal function in MATLAB (version R2015a, MathWorks, Natick, MA, USA). The upper and lower limits of detection (*ULOD* and *LLOD*, respectively) used to calculate the dynamic range (*ULOD–LLOD*) for each glucose challenge were calculated by using the 3-σ method described elsewhere [34]. The sensitivity was calculated by dividing the difference in phosphorescence lifetime values at the *ULOD* and *LLOD* by the dynamic range. All statistical tests were performed using GraphPad Prism (version 7.0a, GraphPad Software, Inc., La Jolla, CA, USA).

### 2.7. Scanning Electron Microscopy of Alginate

Bare and nanofilm-coated alginate microparticles were imaged using a scanning electron microscope (SEM) at 15 kV (JEOL 7500, JEOL USA, Inc., Peabody, MA, USA). Samples were prepared by pipetting 2 μL of a 1:20 dilute solution of microparticles onto a silicon wafer and drying in a vacuum desiccator overnight. The samples were sputter coated with Pd/Pt (2.5 nm) prior to image acquisition.

### 2.8. Darkfield and Hyperspectral Imaging of AnA Hydrogels

AnA hydrogels (4 mm biopsy punch) were used for darkfield and hyperspectral imaging using a research grade optical microscope (BX51, 60x oil, variable NA iris, aluminum quartz halogen source lamp, Olympus, Waltham, MA, USA) equipped with an enhanced darkfield illumination condenser system. Hyperspectral image acquisition was obtained with a diffraction grating hyperspectral imager, integrated CCD, and line scan or “pushbroom” system. CytoViva software (customized ENVI hyperspectral image software, CytoViva, Inc., Auburn, AL, USA) was used for all image processing and nanoscale analysis.

## 3. Results and Discussion

### 3.1. Characterization of the AnA Hydrogels

Figure 1a,b illustrate an alginate microparticle, synthesized by a water-in-oil emulsion method, containing PdTCPP, GOx, and Cat before and after LbL nanofilm deposition of 10 bilayers of PAH and PSS (denoted as [PAH/PSS]_10_). The bare and coated microparticles were imaged to reveal morphological differences using SEM, as shown in Figure 1d,e, respectively. The textured surface of the uncoated microparticles resulted from residual CaCl_2_ salt but was much smoother than the LbL-coated spheres, which exhibit increased surface roughness. After characterization of the alginate microparticles, the AnA hydrogel (Figure 1c) was fabricated by the internal gelation method described above. Darkfield microscopy of the AnA hydrogel (Figure 1f) shows individual alginate microparticles distributed throughout the 1.5 mm thick alginate hydrogel. The reflectance spectrum and hyperspectral imaging of Figure 1f showed an absence of signal in areas of the hydrogel where there was an absence of sensing microdomains, indicating the confinement of PdTCPP to the nanofilm bound microparticles (Figure S1).

### 3.2. AnA Hydrogel Response

Following synthesis and characterization of the alginate microparticles and AnA hydrogels, performance of the full system was evaluated. The sensing capability of this system is governed by the following well-known enzymatic reaction in the presence of GOx: glucose + O_2_ + H_2_O → gluconic acid + H_2_O_2_. Glucose and molecular oxygen diffuse into the sensing material, and glucose is catalytically converted into gluconic acid and hydrogen peroxide by the oxidoreductase enzyme GOx. This reaction results in the consumption of and decrease in local glucose and oxygen concentrations. The co-immobilized phosphorescent porphyrin dye (PdTCPP) is collisionally quenched by molecular oxygen, resulting in phosphorescence intensities and lifetimes that are inversely proportional to the local oxygen concentration [35]. As oxygen decreases, PdTCPP is quenched less; with the co-substrate reaction scheme, this results in increasing phosphorescence lifetimes that are directly correlated with glucose concentrations within the AnA hydrogel. As a result, the systems employing enzymatic AnA hydrogels are flux-based, where performance relies on the ability to control relative diffusion of oxygen and glucose.

In order to tune the sensitivity of the AnA hydrogels under different oxygen conditions, we aimed to control the nature of the diffusion-limiting coatings on the surface of the alginate microparticle. Glutaraldehyde crosslinking of the amine groups on PAH has been shown as an effective method to significantly reduce glucose permeation; therefore, AnA hydrogels containing alginate microparticles without and with crosslinking were synthesized [28]. We quantitatively assessed the effect that crosslinking has on the diffusion of glucose across planar Anapore filters containing 10 bilayers of PAH/PSS and found a 179% decrease in the glucose permeation rate when crosslinking was introduced (Figure 2a). Next, we evaluated the optical phosphorescence lifetime response when oxygen concentrations were varied by using the Stern Volmer relationship: *τ_0_/τ* = 1 + *K_SV_*[O_2_]. The normalized phosphorescence lifetime (*τ_0_/τ*) was plotted against oxygen concentration ([O_2_]) in order to calculate the Stern Volmer quenching constant (*K_SV_*), summarized in Table 1 below. We found no statistical difference between AnA hydrogels without and with crosslinking at oxygen levels lower than 115 μM (*p* > 0.05, Figure 2b), which represents the expected operating conditions (<100 μM [O_2_]) for an in vivo implantable glucose bioresponsive material. With this system, we control the analyte flux only, while the oxygen concentration remains unperturbed; this is especially key for environments that are limited in oxygen supply.

**Figure 3:**
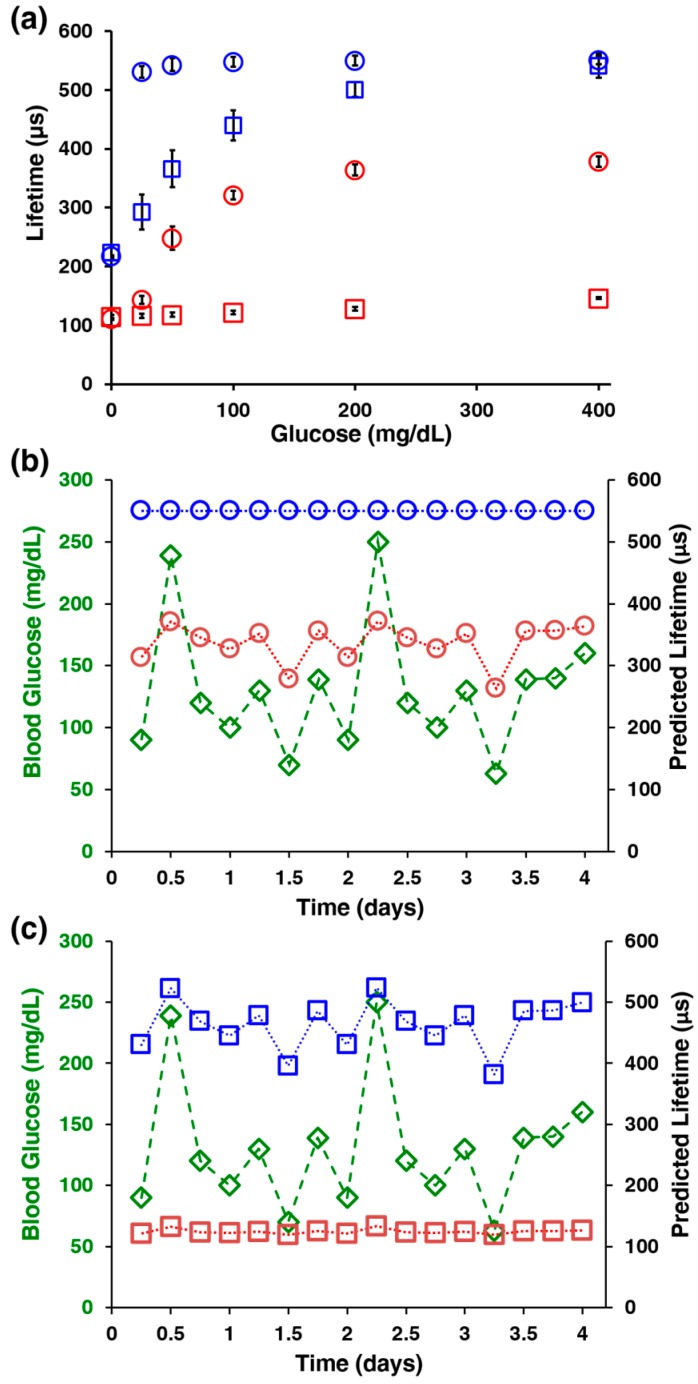
Calibration and performance stability of AnA hydrogels. (**a**) AnA hydrogel response to varying glucose concentration (0–400 mg/dL) for non-crosslinked (blue ○ or red ○, ambient and low oxygen, respectively) and crosslinked (blue □ or red □, ambient and low oxygen, respectively) microparticles. Error bars represent 95% confidence intervals for three separate samples of the same formulation; (**b**,**c**) Simulated blood glucose levels (green ◊, left axis) collected over time and the predicted response of each AnA hydrogel (blue and red, right axis). The predicted values indicate estimated responses from the calibration of materials containing either non-crosslinked (blue ○ or red ○, ambient and low oxygen, respectively) or crosslinked (blue □ or red □, ambient and low oxygen, respectively) microparticles.

Figure 3a is a graph of the phosphorescence lifetime response to glucose concentration changes, which clearly presents the biosensing capability of this system. The AnA hydrogels were tested at physiologically relevant glucose concentrations (0–400 mg/dL) and at two different oxygen levels, ambient (206.8 μM) and interstitial (70 μM). Calibration curves were generated for each response, and the *ULOD* and *LLOD* were calculated in order to determine the dynamic range and sensitivity for each AnA hydrogel. At ambient conditions, the AnA hydrogels containing non-crosslinked microparticles respond over a wide dynamic range of 5.7–330 mg/dL with a sensitivity of 0.80 ± 0.11 μs·dL·mg^−1^. In contrast, AnA hydrogels containing crosslinked LbL coated microparticles under ambient conditions exhibit only a slight increase in the optical response with phosphorescence lifetimes of 115–150 μs correlating to a dynamic range of 87–350 mg/dL and a low sensitivity of 0.075 ± 0.013 μs·dL·mg^−1^ when exposed to glucose. This difference observed at ambient conditions is due to an imbalance in the analyte diffusion kinetics, where the low glucose flux relative to oxygen flux results in a lack of sensitivity when using crosslinked LbL coated microparticles within the AnA hydrogels. Specifically, glucose diffusion through AnA hydrogels with crosslinked microparticles is so slow relative to oxygen that the reaction does not effectively deplete oxygen locally. At low oxygen levels, however, a decrease in glucose diffusion is necessary in order to prevent saturation of the signal. AnA hydrogels containing non-crosslinked particles immediately saturated when exposed to the lowest concentration of glucose. Alternatively, AnA hydrogels containing crosslinked microparticles have a linear response at physiologically-relevant glucose and oxygen concentrations with a dynamic range of 2.6–350 mg/dL and sensitivity of 0.97 ± 0.054 μs·dL·mg^−1^. The sensing figures of merit are summarized in Table 1 for AnA hydrogels containing either non-crosslinked or crosslinked microparticles at ambient and low oxygen concentrations. These results demonstrate the ability to control the desired optical response by carefully engineering the diffusion-limiting coating on alginate microparticles; this is extremely important when translating this bioresponsive composite hydrogel design to in vivo applications. We note some of our parallel efforts specifically address some of the questions pertaining to subcutaneous deployment of such responsive materials. Specifically, we have performed studies with live subjects (animal and human models) to assess the potential for successful in vivo application of similar materials. These comparable sensing systems consist of implantable hydrogel-based bioresponsive materials with longer-wavelength benzoporphyrin phosphors (with red-light excitation, compared to the green-excited phosphors used here) along with a complementary phosphorescence detection instrument with red LED and filters that match the long wavelength emission [23,36]. These studies describe a strategy that appears effective for in vivo use and should be applicable to the AnA hydrogels described here, but they will similarly require a shift to longer-wavelength phosphors to enable deeper tissue penetration.

To better understand the AnA hydrogel performance and further underscore the necessity to tune the sensitivity and dynamic range, we compared a numerical simulation of glucose finger prick measurements with predicted AnA hydrogel response values. Figure 3b,c present graphs of reference glucose values overlaid on a plot of predicted AnA hydrogel response values that would be expected from non-crosslinked or crosslinked microparticles calibrated and operated at ambient and low oxygen concentrations. The glucose values represent measurements from finger prick blood glucose measurements. The predicted optical response values were extrapolated from the calibration curves generated from Figure 3a and are associated with each individual glucose measurement. Figure 3b shows that the incorporation of non-crosslinked microparticles at ambient oxygen concentrations correlates relatively strongly with the simulated glucose values (*r* = 0.810), indicating that the oxygen concentrations at ambient conditions are high enough that they do not necessitate an increased diffusion barrier and decrease in analyte flux. Although AnA hydrogels containing crosslinked microparticles also correlate remarkably well with glucose when tested at ambient oxygen conditions (*r* = 0.998, Figure 3b), the sensitivity is too low to distinguish between the phosphorescence lifetime values for most practical applications. Alternatively, the AnA hydrogels with non-crosslinked microparticles tested at low oxygen conditions generate a constant, saturated response; here, no correlation between predicted phosphorescence lifetime values and input glucose values is expected. Crosslinked microparticles are essential for a robust response at low oxygen levels; these predictions show a dramatically improved correlation with reference glucose values (*r* = 0.904, Figure 3c).

### 3.3. AnA Hydrogel Stability

**Figure 4:**
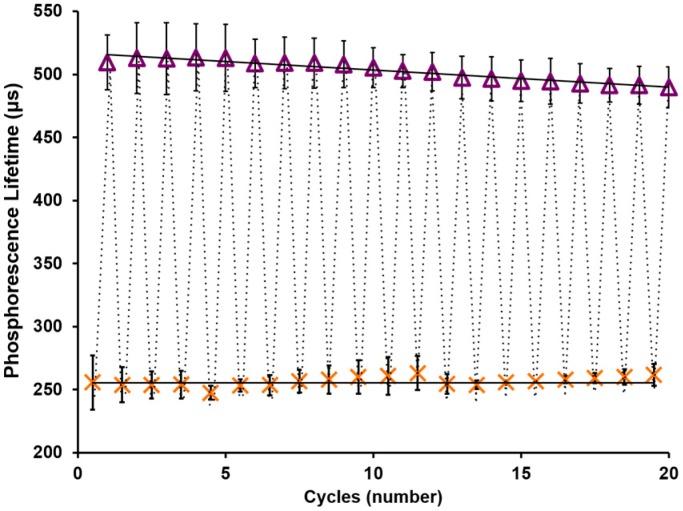
Stability of the AnA hydrogel. Cyclic testing of the AnA hydrogel consisting of crosslinked microparticles tested at interstitial oxygen levels over 2 days. Glucose was varied between 0 mg/dL (orange X) and 400 mg/dL (purple ∆). Error bars represent 95% confidence intervals for three separate samples.

Enzymatic biosensing materials are often degraded over time due to various mechanisms, including spontaneous enzyme denaturation and peroxide poisoning [37,38]. Catalase is an enzyme found in many organisms that is utilized for the catalysis of hydrogen peroxide into water and oxygen and has been incorporated into enzymatic glucose responsive hydrogels to mitigate peroxide poisoning [39,40]. By co-immobilizing catalase with glucose oxidase in our microparticles and using alginate as an enzyme immobilization matrix, the stability was expected to increase. Indeed, Figure 4 shows the remarkable stability of the AnA hydrogel containing crosslinked alginate microparticles tested at an interstitial oxygen concentration (70 μM [O_2_]). The hydrogels were tested repeatedly over the course of two days at the extreme physiological values of 0 and 400 mg/dL. The phosphorescence lifetime data points for each glucose concentration (0 and then 400 mg/dL) were fit with a linear regression line. Although there appears to be a slight increase and decrease in the phosphorescence lifetime values at 0 and 400 mg/dL, respectively, there was no statistically significant difference in the optical response from start to finish at either of the two concentrations studied (*p* > 0.05). Using linear regression to predict the rate of degradation, it was estimated that the AnA hydrogel when exposed to these extreme, cyclic glucose concentrations would lose 50% of the optical signal (phosphorescence lifetime, ~383 µs) at cycle 173; this translates to a functional working lifespan of ~2 weeks.

## 4. Conclusions

Alginate hydrogels embedded with alginate microparticles containing oxygen-sensitive phosphors and oxidoreductase enzymes were demonstrated as stable optical bioresponsive materials. This combination offers a number of attractive features. In terms of performance, these materials exhibit precisely-controlled diffusion of glucose and oxygen into the discrete biosensing domains, which is necessary for accurate sensing at low, physiologically relevant oxygen concentrations. The AnA hydrogels were designed for in vivo sensing applications where long-term stability is necessary; here, we showed remarkable stability after two days of cyclic exposure to extreme glucose concentrations. These results translate to approximately two weeks of stability if exposed to extreme analyte concentrations, further supporting the possibility for use as an implantable sensing material. The stability is attributed to the supportive environment of the alginate microparticles wherein the enzyme is physically trapped. This demonstration of effective control of sensitivity and dynamic range by adjusting the properties of nanofilm coatings on microscale inclusions opens the door for applications of a new class of engineered materials for sensing. The same approach will be applicable when adapting these materials for sensing with other oxidoreductase enzymes for the monitoring of different chronic conditions. Future work will focus on multianalyte analysis and in vivo sensing studies in animal models, where the additional benefit of the modular sensing domains and the biocompatible bulk hydrogel is expected to enhance host acceptance.

## Figures and Tables

**Table 1:** Figures of merit calculated for AnA hydrogels with and without crosslinked microparticles at ambient and low oxygen conditions ^a^.

	Non-Crosslinked	Crosslinked
τ_0_ (μs)	603 ± 16.3	588 ± 22.3
*K_sv_* (μM^−1^·O_2_) × 10^−2^	1.9 ± 0.072	2.3 ± 0.017
**Ambient O_2_**		
Dynamic Range (mg/dL)	5.7–330	87–350
Sensitivity (μs × dL/mg)	0.80 ± 0.11	0.075 ± 0.013
**Low O_2_**		
Dynamic Range (mg/dL)	^b^---	2.6–350
Sensitivity (μs × dL/mg)	^b^---	0.97 ± 0.054

^a^ Data is calculated based off of three separate AnA hydrogels from the same formulation with 95% confidence intervals. ^b^ AnA hydrogel saturated immediately after first glucose concentration; Metrics not determined.

## Supplementary Materials

The following is available online at www.mdpi.com/2079-6374/7/1/8/s1, Figure S1: Reflectance Spectrum and Hyperspectral Imaging.
