# Supplementary material for: Composite Hydrogels with Engineered Microdomains for Optical Glucose Sensing at Low Oxygen Conditions
Source: Biosensors (Basel). 2017 Jan 22;7(1):8. doi: 10.3390/bios7010008 (PMC5371781; doi:10.3390/bios7010008)
Supplement: Supplementary file 1 [file biosensors-07-00008-s001.pdf]

Biosensors **2017**, *7*, 8, doi:10.3390/bios7010008

Supplementary Materials: Composite Hydrogels with Engineered Microdomains for Optical Glucose Sensing under Low Oxygen Conditions

Lindsey R. Bornhoeft, Aniket Biswas and Michael J. McShane

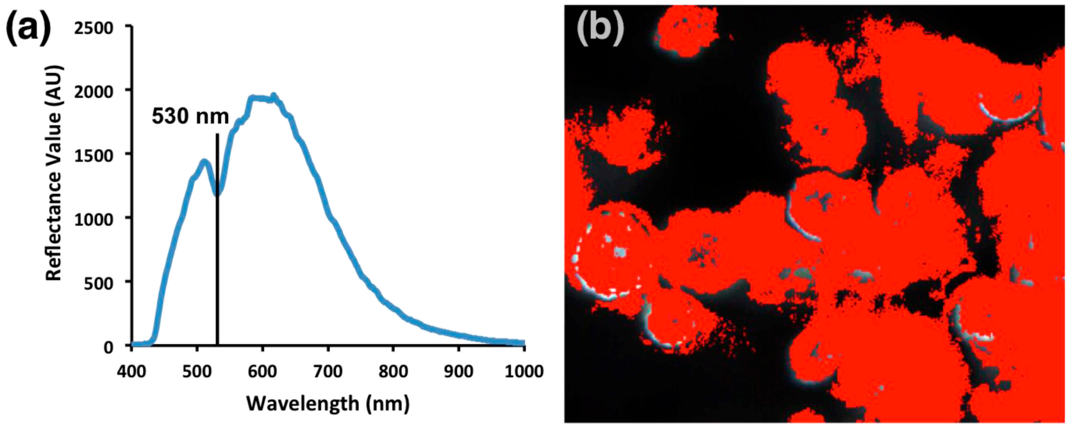

Figure S1: Reflectance spectrum and hyperspectral mapping of PdTCPP within AnA hydrogel. (**a**) Reflectance spectrum of encapsulated PdTCPP embedded within the AnA hydrogel. The decrease in the reflectance value, indicated by a black line at 530 nm, correlates to the excitation wavelength of PdTCPP. (**b**) Hyperspectral map (generated from reflectance spectrum) of a section of the AnA hydrogel with false red color identifying encapsulated PdTCPP at multiple focal planes. Absence of color outside of particles confirms dye is restricted within the nanofilm-coated alginate microparticles and is not present in the matrix. Scale bar = 10 μm.
